# Estimates of the Continuously Publishing Core in the Scientific Workforce

**DOI:** 10.1371/journal.pone.0101698

**Published:** 2014-07-09

**Authors:** John P. A. Ioannidis, Kevin W. Boyack, Richard Klavans

**Affiliations:** 1 Departments of Medicine, Health Research and Policy, and Statistics, Stanford University, Stanford, California, United States of America; 2 SciTech Strategies, Inc., Albuquerque, New Mexico, United States of America; 3 SciTech Strategies, Inc., Berwyn, Pennsylvania, United States of America; Northwestern University, United States of America

## Abstract

**Background:**

The ability of a scientist to maintain a continuous stream of publication may be important, because research requires continuity of effort. However, there is no data on what proportion of scientists manages to publish each and every year over long periods of time.

**Methodology/Principal Findings:**

Using the entire Scopus database, we estimated that there are 15,153,100 publishing scientists (distinct author identifiers) in the period 1996–2011. However, only 150,608 (<1%) of them have published something in each and every year in this 16-year period (uninterrupted, continuous presence [UCP] in the literature). This small core of scientists with UCP are far more cited than others, and they account for 41.7% of all papers in the same period and 87.1% of all papers with >1000 citations in the same period. Skipping even a single year substantially affected the average citation impact. We also studied the birth and death dynamics of membership in this influential UCP core, by imputing and estimating UCP-births and UCP-deaths. We estimated that 16,877 scientists would qualify for UCP-birth in 1997 (no publication in 1996, UCP in 1997–2012) and 9,673 scientists had their UCP-death in 2010. The relative representation of authors with UCP was enriched in Medical Research, in the academic sector and in Europe/North America, while the relative representation of authors without UCP was enriched in the Social Sciences and Humanities, in industry, and in other continents.

**Conclusions:**

The proportion of the scientific workforce that maintains a continuous uninterrupted stream of publications each and every year over many years is very limited, but it accounts for the lion’s share of researchers with high citation impact. This finding may have implications for the structure, stability and vulnerability of the scientific workforce.

## Introduction

The ability of a scientist to maintain a continuous stream of publication has not been well studied. It is well documented that the number of scientists has been growing at a fast pace over time [1], [2]. This growth creates a very large scientific workforce. It would be interesting to evaluate the size and stability of this expanding network. Stability may be more important than the mere size, because uninterrupted, continuous occupation with research may be a major criterion in ensuring achievements for scientists. Scientific investigation may require persistent effort and continuity over many years. Even though major contributions can happen in any single paper, the typical trajectory for a dedicated scientist requires a continuous effort and this may be reflected in a continuous stream of publishing. There are a large number of studies on the age trajectories of scientific careers. The accumulated evidence exhibits substantial variability across disciplines and individuals, and in the relative contributions of scientists at different stages of their career and with different chronologic and academic age [3]–[8]. Different forces, such as changes in creativity/innovation versus building of more collaborations and co-authorship patterns may affect the evolution of scientific productivity, as scientists age. Furthermore, with mounting publish or perish pressure [9], researchers without a continuous publication record often either quit or are forced to quit, since it becomes difficult to attract further funding for their work [10]. At the same time, the potential effect of irregularities in academic lives cannot be ignored. Continuous productivity may be influenced by changing patterns of extensive co-authorship [8] and important differences may exist across scientific disciplines on whether they depend mostly on “core” or “elite” researchers [11]. Some disciplines may have a greater need for cumulative production of information (“cumulative sciences”), while others may work equally well with more sporadic publications of major works that are spaced apart in time.

It would be interesting to obtain empirical evidence and estimates on these patterns and answer some important questions. How many researchers have an uninterrupted, continuous presence (UCP) in the scientific literature over multiple years? Are these scientists more influential than others and do they account for a substantial component of the most highly-cited scientists? Finally, are there some characteristics that separate such researchers with UCP in the scientific literature from others? Here, we aimed to address these questions by analyzing the entire Scopus database in the period 1996–2011.

## Methods

### Database

Using a workable XML version of the entire Scopus database obtained from Elsevier in August 2012, we identified how many authors have published at least one item in each and every year in the 16-year period of 1996–2011. This was the definition of UCP for the covered 16-year period. The unit of analysis adopted throughout the paper is the calendar year, unless specified otherwise. In a sensitivity analysis, we also used a two-year window for the unit of analysis, instead of one-year, i.e. UCP required the publication of at least one item in each and every of the 8 two-year periods in 1996–2001.

Using Scopus, we have previously identified a total of 15,153,100 different author identifiers that have published at least one indexed item in the period 1996–2011 [12]. The database includes all genres of published items, the large predominance though are journal articles. Patents and books are not included in this version of the Scopus database.

### Validation of author identifiers

We used Scopus author identifiers for this project rather than attempting to disambiguate authors on our own. It is possible that some author identifiers that include publications for every year during our 16 year period are cases of polysemy (two or more different authors with the same name grouped under the same author identifier). In most of these cases neither author would qualify for UCP if the author profile were separated correctly. To check for this possibility, we sampled randomly 20 author identifiers with UCP and evaluated their publications by hand to verify that they belonged to a single author publishing continuously over the 16 year period.

We also sampled randomly 20 different author identifiers without UCP to evaluate whether they referred to unique authors who may have published any other Scopus-indexed papers under other author identifiers, and thus may have qualified for UCP if the author identifiers were properly joined. In this case in-depth evaluation meant perusal of all individual publications for all author identifiers with similar first and last names.

### Groups of scientists based on persistence and continuity of publication record

Scientists were grouped into those that had and those who did not have UCP. We also evaluated two other groups. “Skip” authors are defined as those who skipped any year(s) in 1996–2011, and then resumed publication in a subsequent year. This does not count those who have published consecutively for two or more years in the beginning of the 1996–2011 period but not after that (those who did not resume) or at the end of this period but not before that (those who started continuous publication during the time period). “Skip-1” authors are defined as those who would have published in all 16 years had they not skipped only a single year between 1997 and 2010.

### Citation metrics

Citation metrics pertain to the number of citations to papers indexed in Scopus for the years in 1996–2011 and are limited to the citations received until the end of 2011. We measured the number of published items, the citations they had received, and the Hirsch h-index [13] of each author identifier for all Scopus author identifiers and provide descriptive statistics for them for the UCP, no UCP, Skip, and Skip-1 groups.

In order to understand whether differences in citation metrics are due entirely to differences in the number of papers published by authors in each of the four groups, we also performed analyses that show the median citation count and the median h-index for authors in each of the four groups conditioned on the number of papers that they had published over these 16 years. We performed these analyses for the entire database and also separately limited for researchers in Medical Research to use a more homogeneous sample of investigators. These analyses were performed for the entire database using definitions of 1- and 2-year windows for UCP and other categories, and using the 1-year window definition for Medical Research.

### Estimation of birth and death dynamics

One can define the “UCP-birth” and “UCP-death” years of an author as the calendar years that start and end their chain of uninterrupted, continuous annual (or bi-annual, in the 2-yr window case) publications. This does not coincide necessarily with the first and last published paper of each author: some authors that do achieve UCP during their careers may have some skipped years early in their careers (before they start their UCP period); or they may publish some scattered papers over skipped years at the end of their careers (after the end of their UCP period).

We estimated cumulative retention rates of authors by start year. The start year in these analyses was defined as the year in which an author publishes after not publishing the previous year. This is a proxy for the UCP-birth start year. Retention rates for future years were then imputed using year-to-year retention rates (Y2YRR) from the earlier available data. We then estimated the numbers of authors who would be expected to have uninterrupted, continuous presence for 16 years (UCP-16-births) using these imputed Y2YRR rates. For example, the number of authors starting their 16-year UCP in 1999 was estimated by taking the known value from 2011 (n = 23,941 authors publishing each and every year for the 13 years between 1999 and 2011) and multiplying that by the Y2YRR rates for years 14–16 (92% each year).

A similar analysis was carried out using scientists who ceased publication in a particular year. Ending year is defined as the year in which an author published which was immediately followed by a year in which that author did not publish. This is a proxy for the UCP-death end year. UCP rates for earlier years were estimated by extrapolation using Y2YRR from the more recent data.

### Comparison of various characteristics of scientists

We compared the different groups of author identifiers in terms of the main scientific field of the authors. We used a previously developed classification that allocates each paper to a separate scientific discipline and then each author is allocated to a specific discipline depending on what is the most common discipline of the papers he/she has authored – for details see references [14], [15] The resulting 13 scientific fields are Mathematics/Physics, Chemistry, Engineering, Earth Sciences, Biology, Biotechnology, Infectious Disease, Medical Research, Health Sciences, Brain Research, Social Sciences, Humanities, and Computer Sciences/Electrical Engineering. We also compared the different groups of author identifiers in terms of region of Scopus-listed address (North America, Europe, Asia-Pacific, South America, Africa, Middle East, unknown), and sector (academic, hospital, government, industry, society/academy, non-profit, unknown) by in depth evaluation of 10,000 randomly selected author identifiers from each group. Region and sector data are based on our own analysis and curation of the affiliation data associated with the publications of each author. Comparisons of groups used the Fisher-Freeman-Halton exact test with Bonferroni correction of the p-value for the number of comparisons.

### UCP with multiple papers published each and every year

We estimated the number of author identifiers that would fulfill the criteria for UCP during 1996–2011, if the minimum of publications published in each and every year in this period were 2, 3, 4, or 5, instead of just 1.

## Results

A total of 150,608 author identifiers fulfilled our definition of UCP authors. An in-depth evaluation of a random sample of 20 of these author identifiers showed that polysemy (the merging of two or more authors with the same name in the same record) had no major impact on this estimate: all 20 sampled identifiers reflected a specific author who had at least 1 paper published in each and every calendar year.

Authors with UCP are apparently a very small minority of all publishing scientists. Of all the Scopus-indexed scientists, 15,002,492 do not have UCP (see Table 1). Polysemy may result in underestimation of the number of publishing scientists. Conversely, some scientists may have their papers split in two or more different author identifier sets, resulting in overestimation of the total number of publishing scientists. The estimate of authors with UCP is unlikely to be affected substantially, since more than half of the unique author identifiers (58.2%) have papers published only in a single year and the vast majority (91.8%) have papers published only in <8 of the 16 calendar years in the period 1996–2011. Therefore merging potentially split records would not be expected to create many additional authors with UCP. An in-depth evaluation of a random sample of 20 author identifiers without UCP showed that 13 were clearly referring to unique authors who had not published any other Scopus-indexed papers; 2 clearly belonged to authors who had published also papers clustered under 1 or 2 other author identifiers but merging the different records of the same author would not create UCP and would not affect the citation h-index of the larger of the constituent records; and for 5 it was not possible to exclude split records and/or polysemy with perfect certainty, because the names were very common. Overall, the total number of publishing authors with indexed papers in 1996–2011 is probably indeed close to 15 million, meaning that authors with UCP in 1996–2011 are approximately 1% of the total.

**Table 1:** Total published items, total citations and Hirsch h-index for researchers with different patterns of publishing presence in the scientific literature, 1996–2011.

	UCP			Non-UCP			Skip			Skip-1		
Centile	Items	Total Cites	H-index	Items	Total Cites	H-index	Items	Total Cites	H-index	Items	Total Cites	H-index
99%	528	15769	61	53	984	15	46	859	14	207	6132	37
95%	297	8230	45	21	258	8	18	229	7	123	3136	28
90%	220	5647	38	11	109	5	10	98	4	98	2224	24
75%	142	3020	29	4	23	2	3	21	2	71	1297	19
Median	94	1536	21	1	3	1	1	3	1	53	712	14
25%	66	785	15	1	0	0	1	0	0	41	376	11
10%	50	415	11	1	0	0	1	0	0	34	200	8
Mean	122.7	2550	23.1	4.9	58	1.8	4.3	51	1.7	62.1	1069	15.4

The definitions of the 4 groups are: UCP – authors publishing in all 16 years (1996–2011); Non-UCP – authors not publishing in all 16 years (1996–2011); Skip – authors who skipped any of the 16 year(s), excepting those who have published consecutively for two or more years in the beginning of the 1996–2011 period but not after that, and those who published for two or more years at the end of this period but not before that; Skip-1– authors publishing in 15 of the 16 years, with the skipped year between 1997–2010.

The shown total cites and H-index are the average total cites and H-index for each centile in the respective group.

This 1% of scientists defines a very influential core. Table 1 shows that the citation metrics of researchers with UCP are much higher than those of other researchers. While almost three-quarters (n = 110,402, 73.3%) of those with UCP have an h-index of 16 or higher (corresponding to an age-adjusted index m of 1 or higher, a hallmark of a “successful” scientist according to Hirsch), this applies only to 0.96% of those without UCP (n = 144,435), 0.73% (n = 103,475) of “Skip” authors (those who skipped any year(s) in 1996–2011, not counting those who have published consecutively for two or more years in the beginning of the 1996–2011 period but not after that or at the end of this period but not before that), and 43.7% (n = 31,945) of the 73,145 “Skip-1” authors who would have published in all 16 years had they not skipped only a single year between 1997–2010. Similar differences were seen across these groups in the total number of published items and the total citations received. The proportion of authors with an average of at least 20 citations per paper was 38.1%, 10.2%, 10.3%, and 29.6% in the UCP, non-UCP, “Skip”, and “Skip-1” groups, respectively.

When we performed a sensitivity analysis using 2-year windows instead of 1-year windows, the number of scientists who qualified for UCP (at least one item published every two years) non-surprisingly increased three-fold (n = 465181); nevertheless, the newly defined UCP group remained qualitatively different from the other groups, as shown in Table S1.

Overall, the UCP scientists have been involved as authors in 41.7% of the 25,805,462 published items that are indexed by Scopus over these 16 years (Table 2). Of note, as shown in Table 2, the proportion seems to increase slightly until 1999 and decrease slightly afterwards. This may be an artifact, however, of authors who would also qualify for at least 16 years of UCP, if the window of analysis had been extended before 1996 or after 2011. The presence of scientists with UCP is even more impressive in the most highly-cited papers, as they have been involved as authors in 2,778 (87.1%) of the 3,190 papers that received over 1,000 citations.

**Table 2:** Number of published items indexed in Scopus in each calendar year and proportion published by authors with uninterrupted, continuous presence (UCP) from 1996–2011.

Publication year	All items	Number by authors with UCP	%
1996	1134758	487068	42.9
1997	1161780	512768	44.1
1998	1164390	524495	45.0
1999	1166048	537180	46.1
2000	1224001	561538	45.9
2001	1325284	581356	43.9
2002	1374293	600743	43.7
2003	1429751	630560	44.1
2004	1578957	694496	44.0
2005	1755980	752722	42.9
2006	1843420	780745	42.4
2007	1944239	803154	41.3
2008	2020576	821215	40.6
2009	2110248	835968	39.6
2010	2219650	819185	36.9
2011	2352087	807146	34.3
ALL YEARS	25805462	10750339	41.7

**Figure 1:**
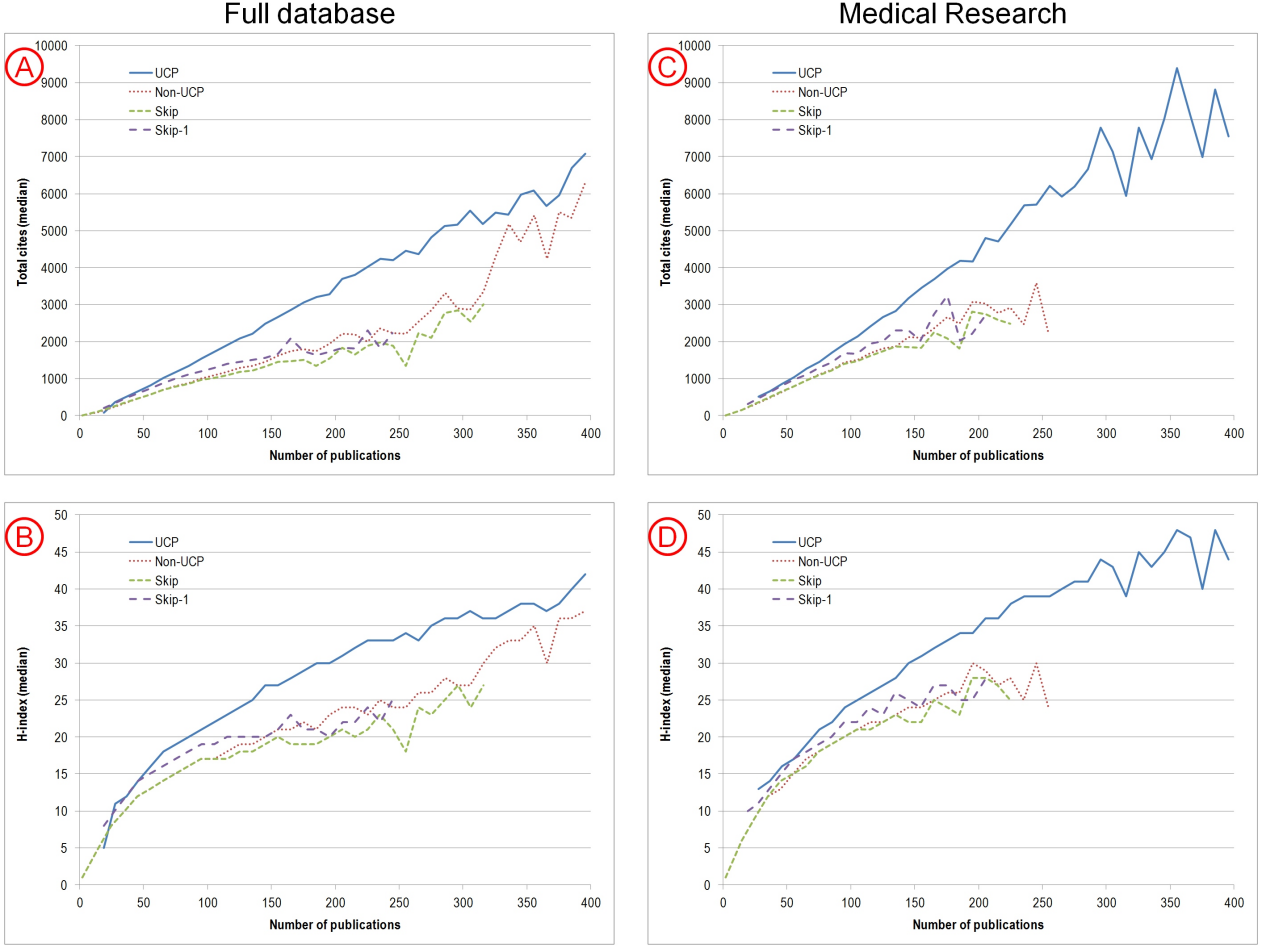
Median citation metrics for authors in the 4 groups according to the total number of papers they authored in 1996–2011: (A) total citation count, (B) h-index, (C) total citation count, limited to Medical Research scientists, (D) h-index, limited to Medical Research scientists. For each of the 4 groups, data are presented for bins of authors spanning 10 papers in the total count of papers (0–10, 11–20, 21–30, 31–40, 41, 50, 51–60, etc), provided that there are at least 50 authors in the bin.

Figure 1 shows the number of median citations (Figure 1A) and median h-index (Figure 1B) for authors in each of the 4 groups conditioned on the total number of papers that they had published. As shown, some of the difference in citation impact in favor of the UCP group is accounted for by the fact that these authors published far more papers. However, even after conditioning on the number of papers, the authors with UCP tend to have higher citation indices when the total number of papers is larger than 50. With fewer than 50 papers total over the 16 years, there is no discernible difference between the UCP and Skip-1 groups. Differences between UCP and Skip-1 groups are also less pronounced at high levels of publication, above 350 papers over the 16 years. These patterns are very similar when we limited the analyses to a specific discipline, Medical Research (Figure 1C and 1D). Moreover, these patterns remained very similar, when sensitivity analyses using the full database were performed using a 2-year window, instead of 1-year window to define UCP (Figure S1).

**Figure 2:**
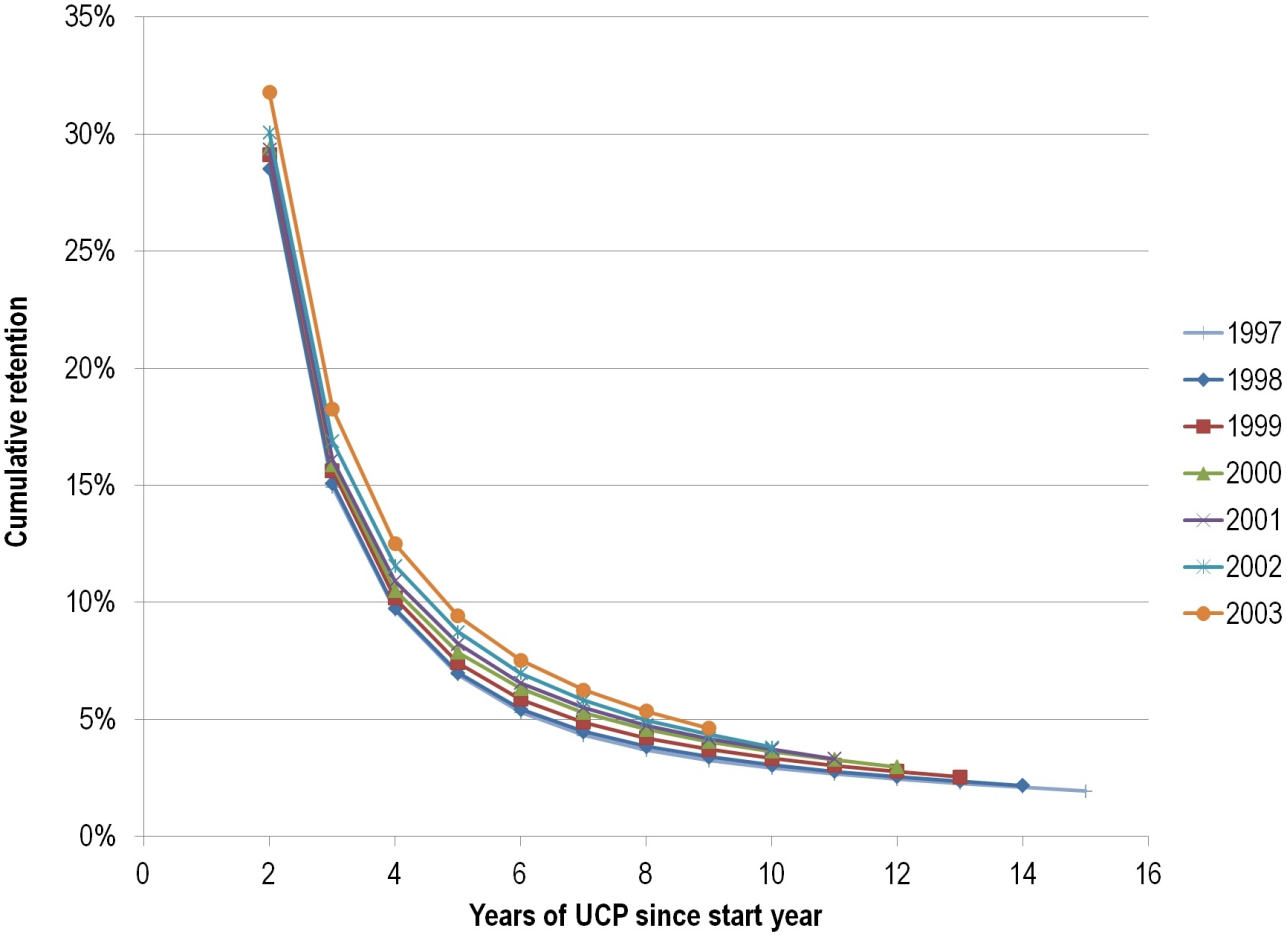
Plotting of the data of Table 3 limited to observed data.

**Figure 3:**
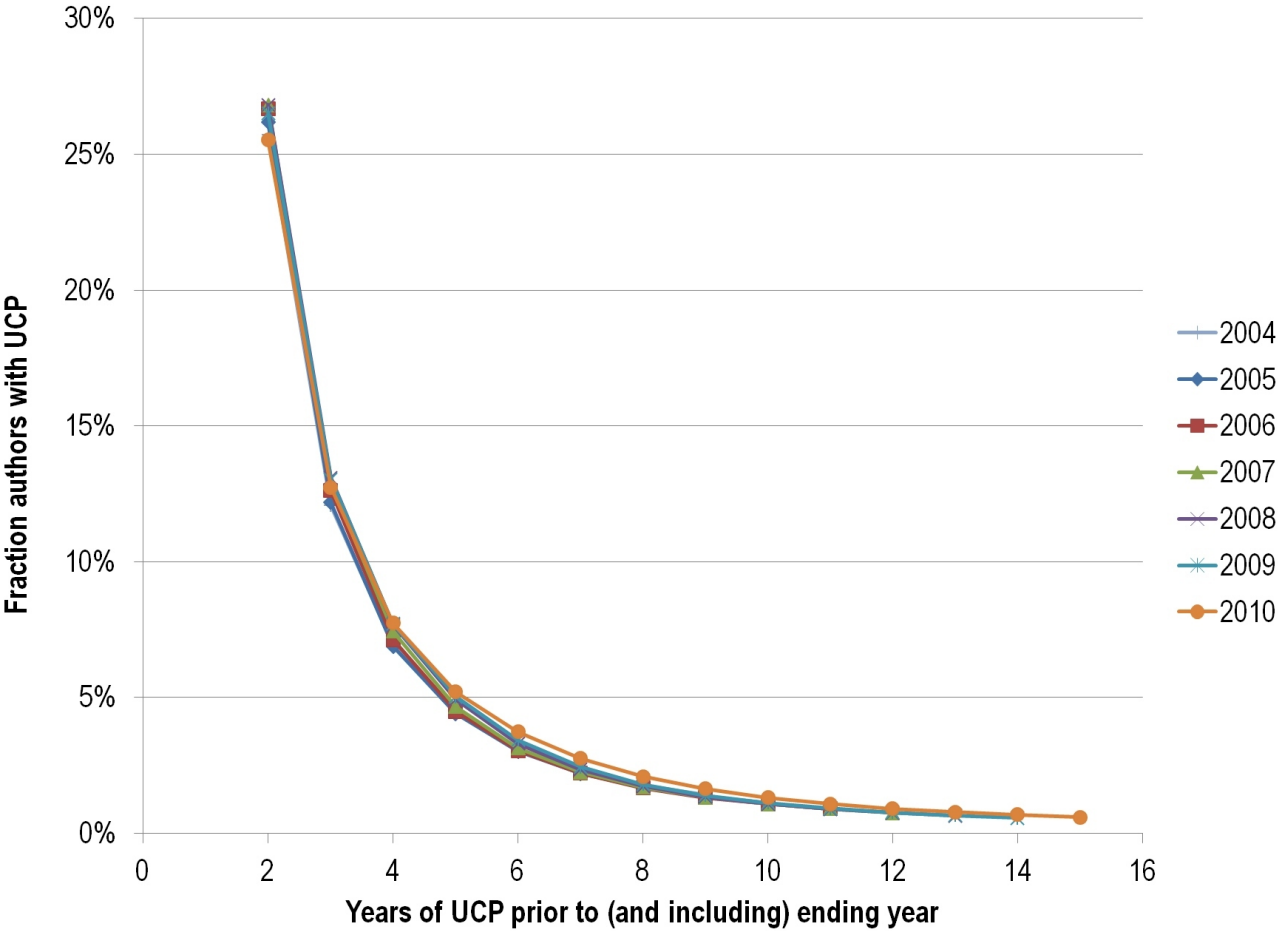
Plotting of the data of Table 4 limited to observed data.

Next, we evaluated the “UCP-birth” and “UCP-death” dynamics of scientists. As shown in more detail in Table 3 and Figure 2, we estimated that 16,877 scientists would qualify for UCP-birth in 1997 (no publication in 1996, UCP in 1997–2012). As an independent verification, in a random sample of 20 of the 18,346 scientists who had uninterrupted, continuous presence in 1997–2011, 18 (90%) also published in 2012. Similarly, we estimated that 9,673 scientists had their UCP-death in 2010 (Table 4 and Figure 3). Raw data are available in Tables S2 and S3. Both the number of scientists who publish in a given year (without having published in the previous one) and the retention rates (the chance of continuing to publish in the next year) appear to be increasing over time.

**Table 3:** Estimated and imputed year-to-year retention rates (Y2YRR) and cumulative retention rates by year for authors based on start year and number of continuous publishing years*.

		Start year						
#Years UCP	Y2YRR	1997	1998	1999	2000	2001	2002	2003
2		28.54%	28.53%	29.12%	29.42%	29.35%	30.06%	31.78%
3		14.93%	15.08%	15.62%	15.87%	16.09%	16.88%	18.25%
4		9.66%	9.73%	10.17%	10.51%	10.91%	11.56%	12.49%
5		6.90%	6.98%	7.40%	7.85%	8.21%	8.73%	9.41%
6		5.30%	5.42%	5.86%	6.32%	6.55%	6.97%	7.53%
7		4.32%	4.47%	4.87%	5.29%	5.50%	5.80%	6.25%
8		3.67%	3.84%	4.20%	4.56%	4.74%	4.97%	5.34%
9		3.24%	3.39%	3.70%	4.04%	4.16%	4.35%	4.62%
10	88%	2.91%	3.05%	3.33%	3.62%	3.73%	3.81%	**4.06%**
11	89%	2.66%	2.77%	3.03%	3.28%	3.31%	**3.39%**	**3.61%**
12	90%	2.44%	2.55%	2.78%	2.96%	**2.98%**	**3.05%**	**3.25%**
13	91%	2.27%	2.36%	2.53%	**2.70%**	**2.71%**	**2.78%**	**2.96%**
14	92%	2.10%	2.17%	**2.33%**	**2.48%**	**2.49%**	**2.56%**	**2.72%**
15	92%	1.93%	**2.00%**	**2.14%**	**2.28%**	**2.29%**	**2.36%**	**2.50%**
16	92%	**1.78%**	**1.84%**	**1.97%**	**2.10%**	**2.11%**	**2.17%**	**2.30%**
UCP-16 births		16877	17402	18657	20840	22301	23934	26562

*Retention rates for future years that have been estimated using year-to-year retention rates (Y2YRR) from the earlier data are shown in bold type. Estimated (by extrapolation) numbers of authors who will have uninterrupted, continuous presence for 16 years (UCP-16-births) are listed at the bottom by start year.

**Table 4:** Estimated and imputed year-to-year retention rates (Y2YRR) and cumulative UCP rates for authors based on ending year and number of continuous publishing years previous to ending*.

		Ending year						
# Years UCP	Y2YRR	2004	2005	2006	2007	2008	2009	2010
2		25.72%	26.18%	26.69%	26.82%	26.82%	26.52%	25.53%
3		12.06%	12.18%	12.62%	12.91%	13.09%	13.04%	12.73%
4		6.94%	6.89%	7.13%	7.44%	7.68%	7.71%	7.75%
5		4.44%	4.41%	4.48%	4.66%	4.92%	5.03%	5.21%
6		3.12%	3.01%	3.04%	3.14%	3.30%	3.44%	3.73%
7		2.30%	2.20%	2.21%	2.25%	2.34%	2.44%	2.76%
8		1.78%	1.69%	1.67%	1.69%	1.74%	1.81%	2.08%
9		1.42%	1.36%	1.32%	1.33%	1.34%	1.40%	1.64%
10	80%	**1.13%**	1.11%	1.08%	1.08%	1.08%	1.12%	1.31%
11	82%	**0.93%**	**0.91%**	0.90%	0.90%	0.89%	0.92%	1.08%
12	84%	**0.78%**	**0.76%**	**0.76%**	0.76%	0.76%	0.77%	0.91%
13	86%	**0.67%**	**0.66%**	**0.65%**	**0.65%**	0.65%	0.66%	0.78%
14	87%	**0.58%**	**0.57%**	**0.57%**	**0.57%**	**0.56%**	0.57%	0.68%
15	87%	**0.51%**	**0.50%**	**0.49%**	**0.49%**	**0.49%**	**0.50%**	0.60%
16	87%	**0.44%**	**0.43%**	**0.43%**	**0.43%**	**0.43%**	**0.43%**	**0.52%**
UCP-16 deaths		5051	5445	5743	6181	6542	7145	9673

*UCP rates for earlier years that have been estimated by extrapolation using year-to-year retention rates (Y2YRR) from the more recent data are shown in bold type. Estimated (by extrapolation) numbers of authors who ceased publishing in the ending year after having published continuously for 16 years are listed at the bottom of the table.

Based on more in-depth evaluation of random samples of 10,000 researchers from each group (Table 5), the relative proportion of UCP authors across scientific disciplines is different than the respective distribution for non-UCP authors (p<0.001). The presence of the UCP pattern is relatively enriched in Medical Research, but also in Mathematics/Physics and Chemistry, while the presence of the non-UCP pattern is relatively enriched in Social Sciences and Humanities (the UCP pattern is practically non-existent in the Humanities), as well as Engineering and Computer Sciences/Electrical Engineering. Moreover, the UCP pattern differs according to geographical region and sector where scientists work (p<0.001 for both). Specifically, the presence of the UCP pattern is enriched predominantly in Europe and less so in North America and the non-UCP pattern is relatively enriched in other parts of the world; and the UCP pattern is enriched predominantly in the academic, government, society/academy, and non-profit sectors, while the non-UCP pattern is relatively enriched in the hospital sector and strongly so in the industry sector. The Skip group had similar characteristics to the entire non-UCP group and the Skip-1 group largely resembled in its characteristics the UCP group (Table 5).

**Table 5:** Characteristics of researchers with different publication patterns, 1996–2011, based on n = 10,000 randomly sampled authors for each pattern.

	UCP	Non-UCP	Skip	Skip-1
Main Field				
Mathematics/Physics	15.7%	5.7%	5.3%	13.7%
Chemistry	12.0%	8.9%	8.6%	10.3%
Engineering	5.7%	9.6%	9.9%	6.9%
Earth Sciences	2.2%	2.2%	2.1%	3.4%
Biology	4.8%	6.0%	5.7%	6.5%
Biotechnology	2.0%	1.7%	1.6%	2.0%
Infectious Disease	4.7%	4.8%	4.9%	5.5%
Medical Research	33.0%	21.0%	21.0%	30.2%
Health Sciences	4.7%	7.1%	7.7%	5.2%
Brain Research	6.7%	5.1%	5.0%	6.9%
Social Sciences	1.5%	5.7%	6.4%	2.4%
Humanities	0.0%	0.3%	0.3%	0.0%
Computer Sciences/Electrical Engineering	6.9%	9.7%	9.1%	6.9%
Unknown*		12.4%	12.6%	
Region				
North America	31.8%	21.8%	22.4%	30.8%
Europe	42.7%	26.0%	26.3%	42.1%
Asia-Pacific	21.2%	27.2%	25.9%	20.5%
South America	1.4%	3.2%	3.3%	2.5%
Middle East	1.6%	2.9%	2.7%	2.1%
Africa	0.4%	1.5%	1.6%	0.9%
Unknown**	0.9%	17.4%	17.7%	1.1%
Sector				
Academic	76.3%	56.8%	56.2%	72.7%
Hospital	4.2%	6.5%	6.9%	5.1%
Government	11.5%	8.0%	8.1%	11.7%
Industry	1.5%	6.5%	6.3%	2.9%
Society/Academy	2.3%	1.6%	1.4%	2.5%
Non-profit	1.5%	0.7%	0.5%	1.4%
Unknown**	2.6%	20.0%	20.7%	3.7%

*Only those papers for which can be linked to others through citation are classified. Thus, the main field for some authors with only one indexed publication cannot be determined.

**Both region and sector information are calculated based on an incomplete list of institutions. All major institutions (those publishing at least 50 papers per year) are accounted for. However, region, and sector information have not been curated for smaller institutions. Thus, the region and sector information for many authors cannot be determined for these less prolific institutions.

Reasons behind the differences observed here between UCP and other groups by discipline, region, and sector cannot be explained using these data alone. Nevertheless, they provide a seed for hypotheses that could be tested using additional combinations of data. For example, enrichment of the UCP pattern in Medical Research, Physics, and Chemistry may correlate with the relatively high infrastructure (e.g., long term grants, capitalized equipment) level associated with these disciplines. In contrast, presence of the non-UCP pattern in Computer Science and Social Sciences may correlate with the relative absence of such infrastructure. Additional data are required to test hypotheses such as this.

The number of scientists who published more than just a single paper in each and every year in 1996–2011 is much smaller than the already small UCP group, as defined above. There were 68,221 scientists publishing at least 2 papers each and every year, 37,953 publishing at least 3, 23,342 publishing at least 4, 15,464 publishing at least 5, and only 3,269 scientists publishing at least 10 papers each and every year in 1996–2011.

## Discussion

Our evaluation of the entire Scopus database for the period 1996–2011 shows that, overall, only a very small fraction of researchers (<1% of the over 15 million publishing scientists) have an uninterrupted, continuous presence in the scientific literature and these investigators account for the lion’s share of authors who eventually have high citation impact. There is some variability on the relative prevalence of these investigators across different scientific disciplines, geographical regions, and sectors. The concentration of 87% of the most highly-cited papers among ∼1% of scientists represents a heavy-tail phenomenon that is much stronger than the heavy-tail phenomena described for the concentration of influential papers in specific high-profile journals [16] or the concentration of most citations to a relatively modest proportion of papers (80/20 law) [17], [18].

Authors with uninterrupted, continuous presence over all these 16 years eventually had a much higher citation impact than other authors. To some extent this higher impact is generated through a larger volume of published papers. However, the citation impact in the UCP authors goes beyond just publishing more papers. Even after conditioning on the number of papers, the total citations and h-index of their work were higher than those of non-UCP authors; the exception was authors with fewer than 3 papers per year and who did not have any discernible difference in citation impact regardless of whether they had UCP or not.

The vast pool of authors without a continuous presence in the literature probably includes very different categories of people. First, some excellent scientists may intentionally prefer to publish sparingly in the journal literature, especially in the humanities and social sciences where books are a predominant form of communication; however, for most fields of current research, not publishing anything over a year is unlikely to be a desired choice, especially in academic circles, in contrast to industry where other deliverables are more important than publications and for hospital clinicians where patient care is more important than published track records. Second, for many researchers interrupted productivity may reflect life events (e.g., childbearing). Empirical studies have addressed for example gender differences in the continuity of scientific careers [19]. Interrupted productivity may also often reflect limitations and obstacles that scientists face, e.g., insufficient funding or infrastructure or other difficulties that create gaps in their productivity or even lead them to abandon science. Third, many authors may only be ancillary personnel or trainees rather than principal investigators. Fourth, we observed some variability in the prevalence of UCP across scientific disciplines. In the cumulative sciences, such as medical research, that depend on the incremental, continuous accumulation of relatively small bits of information, UCP is highly desirable; conversely, in other disciplines such as the social sciences and humanities, continuous publication on an annual basis may not be as necessary or desirable and many successful scientists may have more sporadically, scattered in time, publications of major works. Scientific disciplines with cumulative profiles however account for the large majority of publishing scientists currently.

Regardless of the exact career qualifications and trajectories of individual authors, our analysis suggests that even though the global scientific workforce is enormous, its continuously publishing core is still limited. Given that there are many thousands of universities and research institutions and each has tens and hundreds of teams and departments, the concentration of ∼150,000 researchers can quickly get rarified. Many teams, departments, or even whole institutions may have none or very few researchers who belong to this core and even fewer who have also considerable impact.

With higher UCP-birth than UCP-death rates, this core is apparently growing, but growth remains small in absolute numbers and thus potentially vulnerable. Moreover, part of the growth may also reflect more extensive indexing of journals that already existed anyhow, rather than genuine growth in the number of continuously productive scientists. This artifact has been detected in previous analyses of the growing number of total articles [20].

Widespread interruption and non-continuity may be a sign of system inefficiency, regardless of whether it reflects mature scientists, ancillary personnel, or aspiring trainees who cannot maintain a continuous presence in the scientific literature. Of course, one should allow for differences across various disciplines and research sectors (e.g. academia versus industry) in interpreting these results. Differences also exist in more granular microenvironments, e.g. on the way tenure is granted in different institutions and fields and whether there is a requirement for continuous productivity once tenure has been granted. Nevertheless, there is mounting evidence that the current scientific enterprise may be focusing more on disciplines that require incremental continuous contributions; this is also confounded by the funding and entrepreneurial support of the scientific endeavor [21] and it furthermore affect norms for the training of young doctoral students [22]–[25]. In many disciplines, doctoral students may be enrolled in high numbers, offering a cheap workforce for materializing resource-intensive incremental research agendas. However, in these cases, the research system may be exploiting the work of millions of young scientists for a number of years without being able to offer continuous, long-term stable investigative careers to the majority of them.

The best course of action in response to this picture can be debated. One option is simply to support further those researchers who succeed into maintaining uninterrupted, continuous presence, since they may be pivotal in generating high-impact science. One possible disadvantage is whether this may lead to further polarization of research in already well-established, readily prolific or conforming [26] lines of investigation. A different approach is to give more opportunities to a wider pool of scientists, especially younger ones, to help them secure continuity of productivity and excellence. Peculiarities related to the needs and aspirations of specific scientific fields and accommodation of life events also need to be considered in any strategic planning. Eventually, the stability and continuity of the publishing scientific workforce may have important implications for the efficiency of science.

## Supporting Information

Figure S1 **Median citation metrics for authors in the 4 groups according to sensitivity analysis considering 2-year window instead of 1-year window.** Compare to Figure 1.
(TIF)

Table S1 **Sensitivity analysis based on 2-year windows instead of 1-year windows: Total published items, total citations and Hirsch h-index for researchers with different patterns of publishing presence in the scientific literature, 1996–2011.**
(DOCX)

Table S2 **Raw data (numbers of authors) used to calculate the cumulative retention rates for authors based on start year and number of continuous publishing years reported in** **Table 3** **.**
(DOCX)

Table S3 **Raw data (numbers of authors) used to calculate the cumulative UCP rates for authors based on ending year and number of continuous publishing years previous to ending reported in** **Table 4** **.**
(DOCX)
