# Supplementary figures and images for: Estimates of the Continuously Publishing Core in the Scientific Workforce
Source: PLoS One. 2014 Jul 9;9(7):e101698. doi: 10.1371/journal.pone.0101698 (PMC4090124; doi:10.1371/journal.pone.0101698)

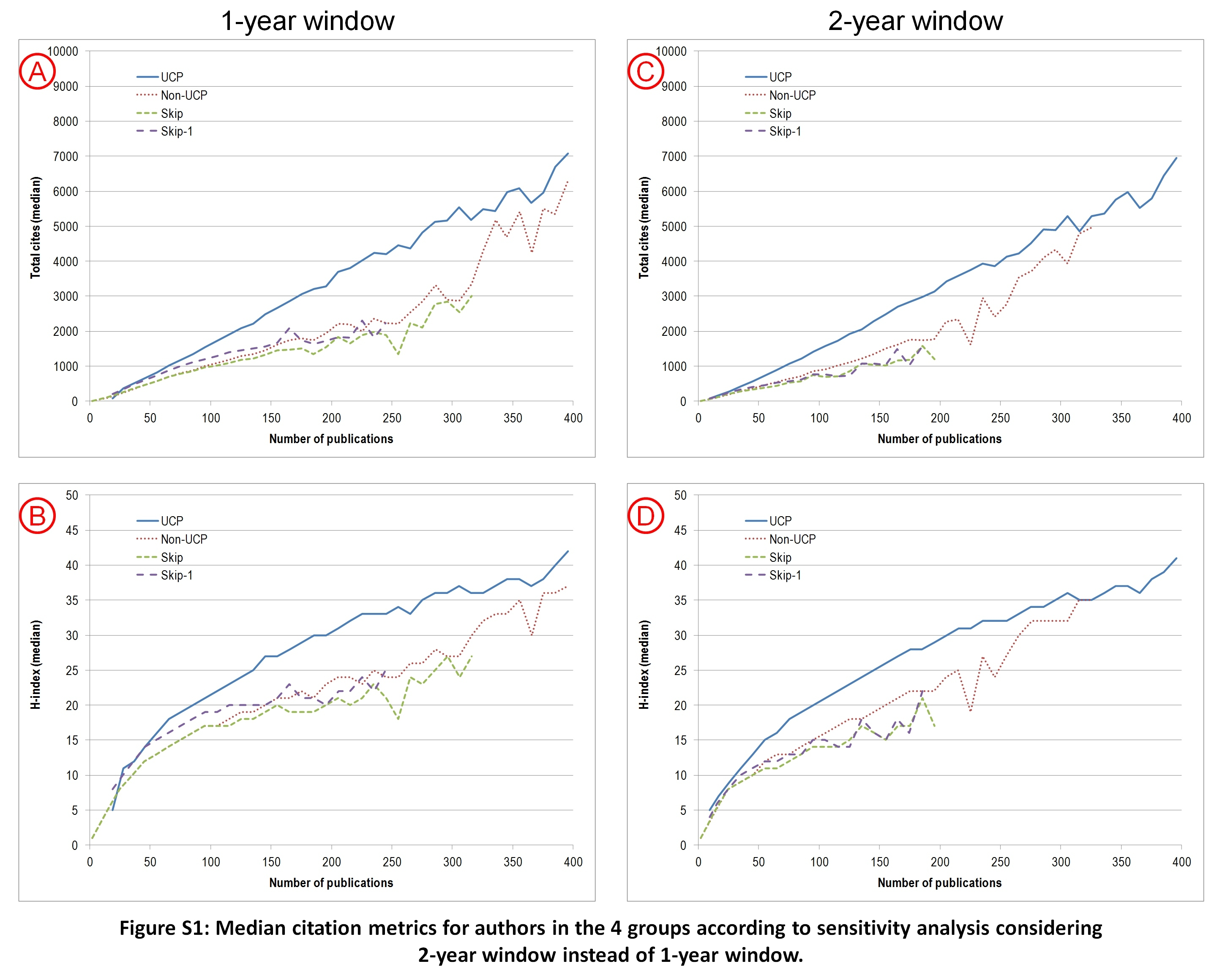

Supplement: Figure S1 — Median citation metrics for authors in the 4 groups according to sensitivity analysis considering 2-year window instead of 1-year window. Compare to Figure 1. (TIF) [file pone.0101698.s001.tif]
