# Supplementary material for: Estimates of the Continuously Publishing Core in the Scientific Workforce
Source: PLoS One. 2014 Jul 9;9(7):e101698. doi: 10.1371/journal.pone.0101698 (PMC4090124; doi:10.1371/journal.pone.0101698)
Supplement: Table S1 — Sensitivity analysis based on 2-year windows instead of 1-year windows: Total published items, total citations and Hirsch h-index for researchers with different patterns of publishing presence in the scientific literature, 1996–2011. (DOCX) [file pone.0101698.s002.docx]

**Table S1. Sensitivity analysis based on 2-year windows instead of 1-year windows: Total published items, total citations and Hirsch h-index for researchers with different patterns of publishing presence in the scientific literature, 1996-2011.**

|  | **UCP** | | | **Non-UCP** | | | **Skip** | | | **Skip-1** | | |
| --- | --- | --- | --- | --- | --- | --- | --- | --- | --- | --- | --- | --- |
| **Centile** | **Items** | **Total Cites** | **H-index** | **Items** | **Total Cites** | **H-index** | **Items** | **Total Cites** | **H-index** | **Items** | **Total Cites** | **H-index** |
| 99% | 369 | 10676 | 50 | 38 | 680 | 12 | 23 | 439 | 9 | 103 | 2608 | 23 |
| 95% | 191 | 4772 | 35 | 16 | 190 | 6 | 10 | 122 | 5 | 58 | 1197 | 16 |
| 90% | 138 | 3061 | 28 | 9 | 86 | 4 | 6 | 56 | 3 | 44 | 814 | 14 |
| 75% | 83 | 1472 | 20 | 3 | 20 | 2 | 2 | 14 | 1 | 31 | 426 | 10 |
| Median | 50 | 663 | 14 | 1 | 3 | 1 | 1 | 2 | 1 | 21 | 201 | 7 |
| 25% | 32 | 290 | 9 | 1 | 0 | 0 | 1 | 0 | 0 | 16 | 88 | 5 |
| 10% | 22 | 128 | 6 | 1 | 0 | 0 | 1 | 0 | 0 | 12 | 37 | 3 |
| Mean | 71.2 | 1337 | 15.9 | 4.0 | 43 | 1.6 | 2.7 | 28 | 1.2 | 26.4 | 365 | 8.2 |

The definitions of the 4 groups are: UCP – authors publishing in all 8 two year intervals between 1996 and 2011; Non-UCP – authors not publishing in all 8 two year intervals between 1996-2011; Skip – authors who skipped any of the 8 two-year intervals(s), excepting those who have published consecutively for two or more years in the beginning of the 1996-2011 period but not after that, and those who published for two or more years at the end of this period but not before that; Skip-1 – authors publishing in 7 of the 8 two-year intervals, with the skipped period between 1998-2009.

The shown total cites and H-index are the average total cites and H-index for each centile in the respective group.
