# Supplementary material for: Estimates of the Continuously Publishing Core in the Scientific Workforce
Source: PLoS One. 2014 Jul 9;9(7):e101698. doi: 10.1371/journal.pone.0101698 (PMC4090124; doi:10.1371/journal.pone.0101698)
Supplement: Table S2 — Raw data (numbers of authors) used to calculate the cumulative retention rates for authors based on start year and number of continuous publishing years reported in Table 3 . (DOCX) [file pone.0101698.s003.docx]

**Table S2: Raw data (numbers of authors) used to calculate the cumulative retention rates for authors based on start year and number of continuous publishing years reported in Table 3.**

|  | **Start year** | | | | | | |
| --- | --- | --- | --- | --- | --- | --- | --- |
| **# Years UCP** | **1997** | **1998** | **1999** | **2000** | **2001** | **2002** | **2003** |
| 1 | 948144 | 945780 | 947072 | 992362 | 1056898 | 1102967 | 1154888 |
| 2 | 270622 | 269804 | 275790 | 291944 | 310223 | 331533 | 367058 |
| 3 | 141519 | 142646 | 147925 | 157500 | 170024 | 186181 | 210821 |
| 4 | 91592 | 92050 | 96311 | 104279 | 115336 | 127531 | 144199 |
| 5 | 65460 | 66057 | 70120 | 77894 | 86802 | 96261 | 108711 |
| 6 | 50216 | 51237 | 55460 | 62718 | 69249 | 76865 | 86906 |
| 7 | 40926 | 42306 | 46138 | 52452 | 58091 | 64014 | 72225 |
| 8 | 34835 | 36304 | 39821 | 45284 | 50089 | 54786 | 61715 |
| 9 | 30713 | 32042 | 35070 | 40065 | 44000 | 47970 | 53337 |
| 10 | 27593 | 28817 | 31549 | 35950 | 39413 | 41977 |  |
| 11 | 25186 | 26215 | 28722 | 32571 | 34983 |  |  |
| 12 | 23172 | 24139 | 26309 | 29409 |  |  |  |
| 13 | 21476 | 22346 | 23941 |  |  |  |  |
| 14 | 19958 | 20534 |  |  |  |  |  |
| 15 | 18346 |  |  |  |  |  |  |
