# Supplementary material for: Estimates of the Continuously Publishing Core in the Scientific Workforce
Source: PLoS One. 2014 Jul 9;9(7):e101698. doi: 10.1371/journal.pone.0101698 (PMC4090124; doi:10.1371/journal.pone.0101698)
Supplement: Table S3 — Raw data (numbers of authors) used to calculate the cumulative UCP rates for authors based on ending year and number of continuous publishing years previous to ending reported in Table 4 . (DOCX) [file pone.0101698.s004.docx]

**Table S3. Raw data (numbers of authors) used to calculate the cumulative UCP rates for authors based on ending year and number of continuous publishing years previous to ending reported in Table 4.**

|  | **Ending year** | | | | | | |
| --- | --- | --- | --- | --- | --- | --- | --- |
| **# Years UCP** | **2004** | **2005** | **2006** | **2007** | **2008** | **2009** | **2010** |
| 1 | 1143757 | 1259377 | 1342305 | 1436956 | 1535391 | 1656852 | 1865037 |
| 2 | 294230 | 329656 | 358232 | 385391 | 411832 | 439330 | 476151 |
| 3 | 137993 | 153368 | 169415 | 185581 | 201001 | 216039 | 237498 |
| 4 | 79343 | 86746 | 95665 | 106842 | 117877 | 127826 | 144470 |
| 5 | 50809 | 55476 | 60177 | 66929 | 75521 | 83372 | 97155 |
| 6 | 35633 | 37923 | 40781 | 45124 | 50646 | 56926 | 69577 |
| 7 | 26311 | 27657 | 29623 | 32273 | 35965 | 40456 | 51474 |
| 8 | 20309 | 21340 | 22455 | 24271 | 26737 | 29946 | 38873 |
| 9 | 16187 | 17078 | 17704 | 19052 | 20648 | 23130 | 30495 |
| 10 |  | 13958 | 14479 | 15531 | 16533 | 18543 | 24502 |
| 11 |  |  | 12072 | 12929 | 13706 | 15164 | 20072 |
| 12 |  |  |  | 10915 | 11630 | 12751 | 16910 |
| 13 |  |  |  |  | 9934 | 10958 | 14542 |
| 14 |  |  |  |  |  | 9440 | 12730 |
| 15 |  |  |  |  |  |  | 11118 |
